# Impact of Functional Feed Additives and Lower Antibiotic Use on Poultry Meat Quality and Consumer Perception

**DOI:** 10.3390/foods15111868

**Published:** 2026-05-25

**Authors:** Abdul Samad, Ayesha Muazzam, AMM Nurul Alam, SoHee Kim, ChanJin Kim, SiHoon An, Young-Hwa Hwang, Seon-Tea Joo

**Affiliations:** 1 Division of Applied Life Science (BK21 Four), Gyeongsang National University, Jinju 52828, Republic of Korea; buzdarabdulsamad@gmail.com (A.S.); ashu2nice@gmail.com (A.M.); alam6059@yahoo.com (A.N.A.); soheelyk@gmail.com (S.K.); ckswls09090@gmail.com (C.K.); ashooon429@gmail.com (S.A.); 2 Institute of Agriculture & Life Science, Gyeongsang National University, Jinju 52828, Republic of Korea; philoria@gnu.ac.kr

**Keywords:** poultry production, functional feed additives, antibiotic alternatives, poultry meat quality, consumer perception

## Abstract

The poultry industry is undergoing a major transition to reduce the use of antibiotics, as a result of the growing concerns about antimicrobial resistance, antibiotic residue in meat and increasingly stringent regulatory policies. This trend has led to an increased interest in functional feed additives as potential alternatives that may support bird health, growth performance and meat quality. There are functional additives, including probiotics, prebiotics, synbiotics, phytogenics, organic acids, enzymes, essential oils, vitamins, minerals and postbiotics, that have shown potential effectiveness in enhancing gut health, nutrient utilization, immunity and disease resistance in poultry. The advantages that are frequently noticed are increased feed conversion ratio, body weight gain, carcass yield and improved meat quality characteristics, such as water-holding capacity, color stability, tenderness, oxidative stability and shelf life. Furthermore, the decrease in the use of antibiotics decreases the risk of residues and also the transmission of antimicrobial resistance genes through the food chain and the environment. Consumer interest in antibiotic-free and naturally raised poultry meat has also led to the emergence of premium market opportunities, where trust, transparency in poultry labelling and perceived safety are key drivers of consumer acceptance. But there are issues yet to be addressed regarding additive efficacy variability, dosage standardization, cost-effectiveness and implementation on farms under different production systems. This review critically evaluates the scientific evidence related to the use of functional feed additives as an alternative to antibiotics in poultry nutrition, focusing on their effects on meat quality, food safety, economic viability, sustainability and consumer perception. Precision nutrition, combinations of synergistic additives, and data-driven feed strategies will be key to future progress to enable profitable and sustainable poultry production.

## 1. Introduction

Over the last few decades, the poultry industry has seen unparalleled growth in the world, with poultry meat production in the world increasing to 133.3 million tons in 2020 [1]. Poultry meat is one of the most affordable and widely consumed sources of protein in the world, supplying necessary protein to billions of people [2]. But the increased production of poultry has brought with it many problems, especially in the use of antibiotics for prophylaxes and growth promotion [3], which contributes to antimicrobial resistance and affects poultry and human health [4]. In the past, antibiotics were added to broiler feed at sub-inhibitory levels to improve feed conversion efficiency, promote growth, and prevent disease outbreaks [5], which creates significant economic returns for the producer. The EU ban on antibiotic growth promoters in 2006 was a turning point, leading to a global transition to alternative strategies [6]. Many countries have now adopted regulations or prohibitions on the use of antibiotics in animal feed for poultry, as a result of consumer demand for “Raised without Antibiotics” (RWA)- and “No Antibiotics Ever” (NAE)-labelled products [5].

In response to the worldwide ban on using antimicrobial growth promoters, the search for natural, safe and sustainable alternatives that can sustain productivity without compromising food safety and public health has intensified [5]. As the poultry industry faces increasing pressure to identify economically viable alternatives to antibiotics that can effectively support growth performance and disease prevention, functional feed additives have emerged as promising solutions. [7]. The most promising alternatives are functional feed additives such as probiotics [8], prebiotics [9], synbiotics [10], organic acids [11], enzymes [12], phytogenics [13] and essential oils [14,15], which have multiple functions and can solve economic and health problems [5]. The global nutraceutical market is expected to reach USD 291.33 billion by 2030, having grown at a compound annual rate of 9.4% between 2022 and 2030, indicating the growing recognition of the industry of these alternatives as a global market [16]. These additives provide producers with the chance to continue to be productive and profitable, while meeting regulatory requirements and consumer demands.

Food safety, nutritional quality and ethical production practices are becoming more important to consumers when purchasing poultry products [17]. Antibiotic residues in poultry meat have serious public health implications, such as allergic reactions, genetic mutations and alteration of normal gut microbiota in consumers [18,19]. Moreover, the presence of antibiotic residues in poultry tissues above Maximum Residue Limits (MRLs) is a significant problem for food safety [20]. Functional feed additives can improve meat quality traits like color, texture, oxidative stability and nutritional composition, while they can also be helpful to decrease the need for antibiotics [21]. The use of these additives helps to maintain consumer confidence and retain the nutritional quality of poultry products [22], contributing to the establishment of premiums for antibiotic-free products.

This review aims to summarize the existing scientific knowledge on functional feed additives in poultry production and critically assess their use as alternatives to antibiotic growth promoters as sustainable feed additives. Moreover, this study also elaborates the mode of action and effectiveness of different classes of feed additives and their impact on growth performance, physiological health and the immune system. While this review primarily focuses on the effects of various feed additives on meat quality and food safety, we also evaluate their economic feasibility and scalability in commercial poultry operations, consumer perceptions and market acceptance, as well as future challenges, opportunities, and research directions for sustainable poultry production.

### Literature Search Strategy and Review Methodology

In poultry production, there have been many years of extensive research on functional feed additives and alternatives to antibiotics. A wide range of scientific literature was gathered from the most important electronic scientific databases, such as Scopus, Web of Science, PubMed and Google Scholar, with a view to obtaining a balanced and comprehensive synthesis of the available evidence. The literature search primarily covered studies published between 2010 and 2026, with some earlier foundational studies included where appropriate. Combinations of keywords like “functional feed additives”, “antibiotic alternatives”, “probiotics”, “prebiotics”, “synbiotics”, “phytogenics”, “organic acids”, “essential oils”, “postbiotics”, “poultry meat quality”, “broiler performance”, “antimicrobial resistance” and “consumer perception” were used in the search terms. The search strategy was modified using Boolean operators (AND/OR).

Original research articles, review papers and authoritative reports on poultry production, meat quality, food safety, antimicrobial resistance and consumer acceptance were considered. Studies on non-poultry species, irrelevant to feed technologies (feed additives) or publications with less scientific value were excluded. To provide reliable and scientifically relevant evidence for discussion in this review, greater weight was given to recent peer-reviewed studies, those with clearly defined experimental designs, and studies published in reputable journals.

## 2. Poultry Industry Dependence on Antibiotics

### 2.1. Historical Use and Growth Promotion Roles

Antibiotics have been used in poultry production systems for over 50 years, mostly as growth promoters and disease preventatives [23]. Low-dose antibiotic administration (subtherapeutic doses) resulted in better feed conversion ratios and increased body weight gain and feed intake in broiler chickens [24]. This practice had a significant impact on the economic viability of intensive poultry production systems, especially in developing nations where antibiotics were still affordable and easily available [25]. Feed formulations were supplemented with antibiotics like bacitracin, tetracyclines and macrolides to modulate the gut microbiota, suppress potential pathogens and increase nutrient absorption [5]. This practice became common, and producers would routinely administer antimicrobials to ensure productivity and minimize losses due to disease.

### 2.2. Disease Prevention and Therapeutic Functions

In addition to growth promotion, antibiotics have been used in poultry to prevent and treat diseases in confined production systems [26]. The intensive nature of modern broiler production makes the situation ideal for the rapid spread of diseases, and therefore, intervention measures need to be implemented to ensure flock health [27]. Antibiotics provide both prophylactic protection against common pathogens and therapeutic treatment for clinical infections caused by bacteria such as *Escherichia coli*, *Salmonella* spp., and *Clostridium perfringens* [5]. In areas where there is a lack of veterinary services and biosecurity infrastructure, routine antibiotic application became a practical solution to maintain production in harsh conditions. But the indiscriminate use of these drugs, particularly their use without veterinary advice or diagnosis, allowed for the rapid emergence and spread of antimicrobial resistance.

### 2.3. Risks of Antibiotic Overuse and Emerging Antimicrobial Resistance

Antimicrobial resistance (AMR) is one of the greatest public health challenges of the 21st century, with livestock production recognized as a significant contributor to its emergence and dissemination. [28,29]. Meanwhile, poultry is a significant driver of AMR [30]. The use of inappropriate antibiotics in poultry production has contributed to the spread and emergence of multi-drug-resistant bacteria in the food chain [31]. Resistant pathogens have been documented in poultry products from various countries, including *E. coli* and *Salmonella* isolates resistant to ampicillin, fluoroquinolones, tetracyclines and other critical antimicrobials [32]. The resistance rates of *Salmonella* to tetracyclines in Southeast Asian countries are 25–95.7% and 13.3–89.5% for penicillin [33]. Mobile genetic elements such as plasmids can spread antimicrobial resistance genes horizontally between bacteria, allowing resistant bacteria to remain in environmental reservoirs, such as poultry manure and farm runoff [34]. Moreover, the presence of antibiotic residues in poultry tissues is a direct health concern to the consumer [35], as in some studies, levels have been found to be more than 10 times higher than the permitted levels [20].

### 2.4. Regulatory Restrictions and Compliance Challenges

AMR regulation has been increasingly tightened worldwide. In 2006, the European Union (EU) banned the use of antibiotic growth promoters (AGPs), which paved the way for a number of countries to do the same [27]. Enforcement and compliance, however, are not consistent, especially in developing economies where antibiotics are still routinely used for economic reasons and there is a lack of regulatory control [31]. Meeting increasingly stringent regulations has imposed significant economic challenges on producers transitioning to new production systems, particularly in low-income regions where profit margins are already limited [36]. Further, there is a lack of transparency in feed supply chains and misleading feed labelling, which makes monitoring and enforcement more difficult, and farmers may not be aware of the presence of antimicrobials in purchased feeds [25].

## 3. Functional Feed Additives as Alternatives

### 3.1. Definition and Classification Framework

According to Chen et al. [37], functional feed additives are bioactive substances deliberately added to animal feed to provide health benefits in addition to nutrition. The additives act on several mechanisms such as modulation of gut microbiota composition, strengthening the intestinal barrier function, stimulating immune responses, and the production of antimicrobial metabolites [38]. The classification of functional additives has changed considerably over the years, and functional additives are now classified as microecological agents (MEAs), such as probiotics, prebiotics, synbiotics and postbiotics [37]. This classification approach overcomes previous nomenclatural ambiguities and allows more precise application strategies in precision livestock farming. Feed additives are different, but they are all complementary, and strategic combination approaches can lead to maximum effectiveness. There are several types of functional feed additives in poultry production that can be divided into groups according to their biological functions, mechanism of action and physiological effect on the host. A summary of the main classes of functional feed additives and their main advantages is given in Table 1.

### 3.2. Probiotics: Mechanism and Efficacy

The most widely studied category of feed additives is probiotics, which are defined as live microorganisms that when consumed by the host provide a health benefit [23]. The most commonly used commercially available probiotic strains for poultry are Lactobacillus species (*L. acidoph-ilus*, *L. plantarum*), Bifidobacterium species and Bacillus species (*B. subtilis*, *B. licheniformis*) [48]. These microorganisms act by competitive exclusion of pathogens, production of antagonistic factors such as bacteriocins and organic acids, and modulation of the intestinal pH [38]. Probiotics increase the production of brush border enzymes and the height of villi, which results in better digestibility and absorption of nutrients and thus better feed conversion ratios and growth performance [49]. Multi-strain probiotic products have been found to be more effective than single-strain probiotics, and the effect of a combination of complementary strains has been shown to be synergistic, enhancing the immunological and performance effects of each strain [50]. Combined probiotic–organic acid supplementation has been shown to result in a body weight gain of 2000 g, a feed conversion ratio of 1.65, 1% mortality and increased microbial diversity (Shannon Index: 4.0), showing overall better performance compared to single treatments in a randomized controlled trial with Cobb 500 broilers [50].

### 3.3. Prebiotics and Their Selective Fermentation

The prebiotics are the components of food that are not digested by the human body and selectively promote the proliferation and metabolic activity of the beneficial microorganisms already present in the gastrointestinal tract [51]. The most commonly studied prebiotic compounds in poultry nutrition are mannan-oligosaccharides (MOS) and fructo-oligosaccharides (FOS) [52]. These substances are not broken down by enzymes in the upper digestive tract and are intact when they reach the hindgut, where they are selectively fermented by beneficial bacteria like *Lactobacillus* and *Bifidobacterium* [38]. This fermentation process produces short-chain fatty acids (butyrate, propionate, acetate), which have several roles, e.g., reducing the pH in the colon to inhibit pathogenic bacteria, supplying energy to the colonocytes, and regulating immune responses [53]. Prebiotic supplementation at 0.1–0.2% of feed has been shown to have a marked effect on carcass characteristics, meat quality and oxidative stability, especially mannan-oligosaccharides, which have been seen to have a significant effect on reducing cholesterol content and improving water-holding capacity [52].

### 3.4. Synbiotics: Strategic Combination Approaches

The strategic association of certain probiotics and suitable prebiotics to create an optimal environment for the growth of beneficial microorganisms is called synbiotics [54]. In synbiotic formulations, the probiotics and prebiotics are not added together, but rather, the selected strains are optimized to ferment the preferred substrates [55]. In several comparative studies, the synergistic effect of well-designed synbiotics has been shown to be greater than the effect of either synbiotic component [50]. This has become a very popular strategy in recent years as the most advanced method for the composition of the gut microbiota. Synbiotics have been shown to enhance feed conversion ratio, growth rate, disease resistance and meat quality parameters in studies, making synbiotics an excellent alternative for producers looking for maximum performance gains [56].

### 3.5. Phytogenic Additives and Herbal Extracts

Essential oils, herbs, botanicals and oleoresins are plant-based compounds that have been identified as having multiple biological activities and are especially promising alternatives, known as phytogenic feed additives (PFAs) [57]. They contain high concentrations of bioactive compounds that have antimicrobial, antioxidant, anti-inflammatory and immunomodulatory properties [58]. Common ingredients used in phytogenic formulations are oregano, thyme, rosemary, turmeric, ginger and garlic, which each possess different bioactive profiles [59]. Studies have shown that phytobiotics can inhibit pathogenic bacteria such as *Salmonella* spp., *Clostridium perfringens* and *E. coli* and stimulate beneficial bacteria [57]. Interestingly, some phytogenic ingredients have antiparasitic properties against *Eimeria* spp. which add extra value in coccidiosis control [59]. In addition to antimicrobial activities, phytogenics stimulate the production of digestive enzymes, improve the morphology of the intestine by increasing the height of the villi and the ratio of villi to crypts, and reduce oxidative stress by activating endogenous anti-oxidant systems [57]. The cost-effectiveness, scalability, and sustainability of plant-based additives, along with their perception as “natural,” which aligns with consumer preferences, make PFAs a preferred choice in many production systems [5].

### 3.6. Organic Acids and Short-Chain Fatty Acid Strategies

Organic acids, particularly short-chain fatty acids (SCFAs) such as butyrate, propionate, and acetate, and medium-chain fatty acids (MCFAs), exert antimicrobial effects through mechanisms that vary according to their pKa values [60]. These acids are dissociated in the acidic conditions of the foregut, creating dissociated molecules that enter bacterial cell membranes and disrupt the pH of the cell, resulting in the death of the bacteria [61]. Organic acids like citric acid, lactic acid, etc., when added to poultry diet, lower the pH of the gastrointestinal tract, which makes it unfavorable for pathogenic bacteria and favorable for acid-tolerant microorganisms [61]. Organic acids have been shown to enhance feed conversion ratio and growth performance and decrease the incidence of digestive disorders when included in feed at 0.5–2% [62]. The synergistic effect of the combination of organic acids, probiotics and prebiotics has been well documented, and combination strategies have been shown to be more effective than single-additive strategies [63].

### 3.7. Enzyme Supplements for Digestive Enhancement

Dietary enzyme supplements, especially those that break down non-starch polysaccharides (NSPs) like xylanase, amylase and protease, can improve the nutritional quality of feed ingredients that are indigestible [61]. NSPs can cause intestinal viscosity and decrease nutrient absorption and growth performance in cereal-based diets, which are widely used in poultry production [61]. Exogenous enzyme supplementation enhances the apparent digestibility of nutrients, enhances the bioavailability of amino acids and minerals, and supports the development of beneficial microbiota [64]. Enzyme supplements have shown special efficacy when used in conjunction with prebiotic and probiotic additives, which help to create a synergic environment for better intestinal function [65].

### 3.8. Essential Oils as Multi-Functional Bioactive Agents

Aromatic plants are rich sources of essential oils that are rich in bioactive compounds with proven antimicrobial, antioxidant and anti-inflammatory properties [66]. Oregano, thyme, rosemary, lavender and blends for specific health issues are common essential oils used in poultry nutrition [67]. The main antimicrobial activities include the disruption of bacterial cell membranes, interference with cellular metabolism and inhibition of production of virulence factors [68]. In particular, essential oils have been found to be effective against gastrointestinal pathogens and parasites, with oregano oil being found to inhibit *E. coli* strains by 97–98% [68]. In addition to their antimicrobial properties, essential oils can improve digestive enzyme activity, increase intestinal barrier function and decrease intestinal inflammation [69]. Essential oils have an antioxidant activity due to their phenolic and terpene contents, which helps to increase the shelf life of meat products by inhibiting lipid oxidation [67].

### 3.9. Minerals and Vitamins as Supportive Nutrients

Certain minerals and vitamins are strategically important to support immune function, antioxidant defense systems and barrier integrity, all of which are essential to resilience in antibiotic-free production systems [70]. Zinc and selenium serve as cofactors for zinc finger proteins and selenoproteins, which are involved in immune regulation and antioxidant defense [71]. The supplementation of *α-tocopherol* (vitamin E) and vitamin C increases anti-oxidant capacity, which helps to prevent tissue damage by oxidative processes and improves meat oxidative stability [72]. The synergistic effects of micromineral fortification, especially in an organic form with improved bioavailability, have been shown with other feed additives [70].

### 3.10. Postbiotics and Novel Emerging Additives

**Figure 1:**
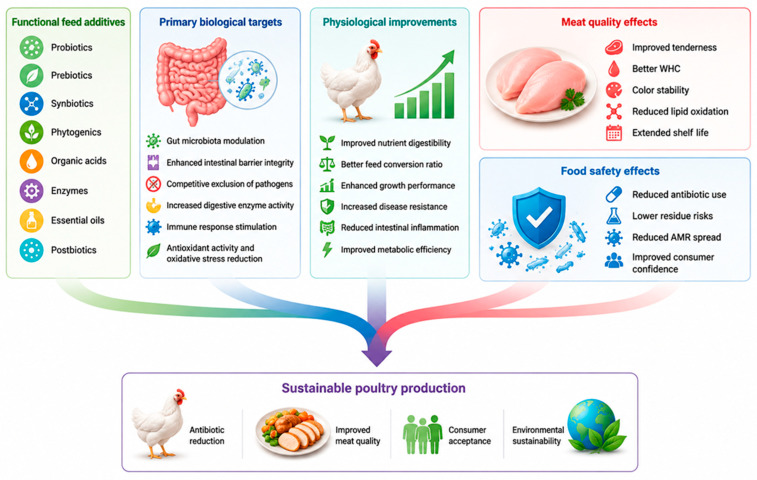
Mechanistic pathways of functional feed additives in poultry production and their effects on health, physiological improvements, food and meat safety and quality, and antibiotic reduction.

Postbiotics are a new category of additives that are made from non-viable inactivated probiotic derivatives and their metabolic by-products and that possess the safety advantages of non-living preparations but also have strong bioactive effects [73]. These products include fermentation metabolites, cell wall fractions and inactivated whole cells that still possess antimicrobial, immunomodulatory and metabolic stimulating properties [74]. New technologies like bacteriophages, antimicrobial peptides (AMPs), hyper-immune egg yolk antibodies (IgY) and genomic medicines hold great promise but need further development to be used on a commercial scale [5]. Another innovative alternative is alginate oligosaccharides derived from tropical seaweeds, which are prebiotic, immunomodulatory, and antioxidant and are comparable or even superior to the effects of conventional antimicrobial growth promoters (AGPs) [75]. Functional feed additives improve poultry health and productivity through multiple interconnected biological mechanisms, including the modulation of intestinal microbiota, enhancement of gut barrier integrity, stimulation of immune responses, improvement of nutrient utilization and reductions in oxidative stress. The major mechanisms associated with functional feed additives and their effects on poultry performance and meat quality are illustrated in Figure 1.

## 4. Effects on Poultry Growth Performance and Health

### 4.1. Feed Conversion Ratio Improvements

Feed conversion ratio (FCR) is an important economic indicator that influences profitability and directly influences the feed requirement for a unit of meat [76]. Many studies have demonstrated the effectiveness of functional feed additives in consistently lowering the FCR, particularly probiotics, prebiotics and synbiotics. In a large scale trial on different classes of additives, probiotics alone reduced FCR during the starter phase (1–10 days) from 1.42 to 1.39 and during the grower phase (10–21 days) from 1.65 to 1.55 when compared with the antibiotic treatments [77]. In extensive 42-day trials, the FCR of the synbiotic supplemented groups was 1.65, whereas the FCR of the controls was 1.72, and this is even more impressive [50]. The improvements in FCR are due to several mechanisms, such as better digestibility of nutrients, less pathogenic bacteria and better intestinal barrier function, which enables better nutrient absorption [64].

### 4.2. Body Weight Gain and Growth Velocity

Body weight gain (BWG) and average daily gain (ADG) are important performance indicators in broiler production that directly affect the length of the production cycle and market readiness [76]. Studies have shown that a well-designed functional feed additive program can achieve growth performance equal to or better than that of an antibiotic program. Bentahar et al. [78] reported that multi-strain probiotic supplementation resulted in body weight gains of about 2787.5 g and 2750.0 g on day 42, whereas the control group had a body weight gain of 2356.8 g [41]. Similarly, Younis et al. [50] conducted a 42-day trial and stated that synbiotic mixtures increased weight gain and health parameters and enhanced microbial diversity. Balanced intestinal microbiota composition is responsible for the improvements in growth performance, which is due to better nutrient utilization efficiency, reduced inflammation and optimized metabolic functioning [38].

### 4.3. Immunity Enhancement and Immune Response Modulation

The immunological effects of functional feed additives are not just about growth promotion but also enhancing the natural resistance and recovery ability of the host to pathogenic challenges [76]. Probiotics induce the synthesis of secretory immunoglobulin A (sIgA) and improve the activity of intraepithelial lymphocytes (IEL) in the intestine, thus boosting the mucosal immune system [38]. Short-chain fatty acids produced during prebiotic fermentation are used as fuel for immune cells and regulate inflammatory responses, thus decreasing pro-inflammatory cytokine production [53]. Immune markers have been measured in studies and consistently show improved responses in additive-treated birds, including increased levels of IL-2 and IL-4, enhanced lymphocyte proliferation and decreased superoxide production [48]. Birds fed with functional additives show a marked increase in the activity of antioxidant enzymes including glutathione peroxidase, superoxide dismutase and catalase, and their antioxidant status is enhanced [58]. These immune-enhancing properties result in better disease resistance, less reliance on antibiotics and greater resistance to production stressors.

### 4.4. Gut Health and Microbiota Modulation

The gastrointestinal microbiota plays an important role in host health, regulating nutrient digestion, immune development and pathogen resistance [79]. Functional feed additives have a strong impact on the composition and activity of the microbiota, thereby creating a favorable microbiota environment. Probiotics have been shown to reduce the number of pathogens like *E. coli* and *Clostridium perfringens* and enhance the populations of beneficial bacteria such as *Lactobacillus*, *Bifidobacterium* and other lactic acid bacteria [77]. Probiotic-supplemented birds have higher alpha diversity of bacteria and more stable microbial community structure, especially when used in combination with complementary prebiotics [50]. The better microbial composition is associated with increased short-chain fatty acid production, better barrier function and decreased intestinal inflammation [53]. Histomorphological analysis reveals that birds fed with additive supplements have better intestinal architecture, characterized by higher villus height, lower crypt depth and better villus-to-crypt ratio, which are all parameters of better absorptive capacity [48].

### 4.5. Disease Resistance and Pathogen Challenge Response

The best indicator of health status is the ability of birds to withstand and overcome disease challenges [80]. This resistance can be greatly improved with functional feed additives under a variety of disease challenges. Controlled *Salmonella* challenge studies have showed synbiotic groups to have significantly better microbial diversity and enhanced resistance after Salmonella challenge, with significantly lower mortality (1%) than that of the control groups (higher mortality) [50]. Some strains of probiotics, such as *Bacillus subtilis*, have been shown to significantly lower the counts of *Clostridium perfringens* after coccidial challenge, and additive-supplemented birds had lower counts of pathogenic bacteria despite the presence of parasites [65]. Mechanisms of disease resistance improvement involve competitive exclusion by direct antagonism of pathogens, production of bacteriocins and metabolites with antimicrobial activity, and increased immune surveillance [64]. Functional additives also allow for a reduction in the metabolic cost of combating infection and for more resources to be directed towards growth and production. Numerous studies have demonstrated the positive effects of functional feed additives on growth performance, feed efficiency, immune modulation and disease resistance in poultry. A summary of representative findings from previous studies is presented in Table 2.

## 5. Impact on Poultry Meat Quality

### 5.1. Carcass Yield and Processing Characteristics

Carcass yield is one of the most important economic indicators that directly affects profitability and resource efficiency [91] and is an important economic parameter. Typically, functional feed additives do not negatively affect carcass yield compared to antibiotic controls, and prebiotic inclusion at 0.2% mannan-oligosaccharide (MOS) has been shown to significantly increase cut up parts yield [52]. Increased meat yield and improved carcass traits have been achieved in multi-strain probiotic supplementation without any adverse effects [92]. The mechanisms involved in yield improvement are increased feed efficiency, decreased inflammatory responses that could affect growth and optimized protein deposition patterns [58]. Importantly, these improvements occur without the residue issues of antibiotic-treated birds.

### 5.2. pH and Water-Holding Capacity

Postmortem pH and water-holding capacity (WHC) of meat are critical factors that affect sensory properties, shelf life and processing suitability [93]. The optimization of pre-slaughter stress and metabolic status by functional additives leads to normal pH decline postmortem, which leads to better functional properties in the meat. Prebiotic supplementation in the range of 0.1–0.2% MOS increased the water-holding capacity and extract release volume in breast and thigh muscles [52]. Meat with better water holding capacity properties was obtained by combining purslane with prebiotics and probiotics, which helped to improve the processing yield and purge losses [94]. These improvements are the downstream effects of the optimized digestive function and diminished inflammation throughout the body due to the optimized digestive function.

### 5.3. Color Stability and Myoglobin Oxidation

Meat color is an important attribute that influences consumer buying decisions and perceived quality and is determined by L* (lightness), a* (redness) and b* (yellowness) values [95]. Bioactive functional feed additives, especially phytogenics and antioxidants, help to maintain color stability and prevent oxidative browning during storage [96]. Phytogenic additives have been found to have a positive effect on the color stability of birds, and the increased antioxidant capacity of the tissue was proposed as the explanation for the higher resistance of myoglobin to oxidation [58]. Probiotics have lower levels of lipid oxidation markers and retain color and palatability after the prolonged storage of meat [94]. The antioxidant properties of the essential oils and plant-based additives, plus the enhanced absorption of antioxidants in the diet and deposition in the meat [67], contribute to the antioxidant properties of the meat itself.

### 5.4. Texture, Tenderness and Sensory Attributes

The textural characteristics of poultry meat (tenderness, juiciness and palatability) are influenced by muscle structure, water-holding capacity, and lipid composition [97]. The addition of postbiotics has been shown to be very beneficial for meat texture, with reduced cooking and drip losses and lower shear force, resulting in better tenderness and juiciness [21]. Probiotics were found to enhance the sensory evaluation parameters of flavor, odor and overall acceptability in birds [21]. The mechanisms responsible for the improvements in texture are optimized protein turnover, decreased muscle fiber degradation, and increased moisture-holding capacity, resulting from improved intestinal health and nutrient absorption [98].

### 5.5. Oxidative Stability and Shelf Life Extension

Lipid oxidation is an important pathway of meat quality degradation that reduces meat shelf life and may produce toxic compounds [99]. Meat oxidative stability and shelf life significantly improved using functional feed additives containing antioxidant compounds [100]. Curci et al. [101] stated that after 7 days of storage, broilers that were fed with essential oil had significantly lower lipid oxidation, as measured by thiobarbituric acid reactive substances (TBARS). Similarly, Bentahar et al. [78] stated that meat samples treated with probiotics from traditional sources (*Levilactobacillus brevis* strains) showed lower TBARS values (0.59–0.60 mg MDA/kg) than a control (0.82 mg MDA/kg). Moreover, Monika et al. [21] stated that the improvement in oxidative stability is associated with increased levels of tissue carotenoids and increased activity of antioxidant enzymes. The increased shelf life of meat from birds with additives gives great economic benefits in terms of minimizing waste and widening markets. Furthermore, improved product stability and reduced spoilage contribute to waste valorization by decreasing post-harvest losses and enhancing the efficient utilization of poultry resources throughout the supply chain [102].

### 5.6. Nutritional Composition and Amino Acid Profiles

Meat is essential to human dietary protein requirements [103,104,105]. Meanwhile, the amino acid composition and nutritional value of the meat are critical determinants of overall meat quality, directly influencing its health benefits, protein functionality and consumer acceptance. Functional feed additives influence nutrient absorption and protein metabolism, thereby optimizing meat composition and improving overall nutritional quality. Bentahar et al. [78] stated that meat protein content was significantly higher in broilers supplemented with multi-strain probiotics (22.50% in the probiotic groups and 21.36% in the control groups) and improved mineral content. Similarly, phytogenic additives have been shown to produce meat with better amino acid profiles and better retention of essential amino acids when fed as diet supplements [58]. Hossain et al. [92] reported that the higher dry matter and nutrient content observed in meat from additive-supplemented birds indicates improved nutrient bioavailability and enhanced metabolic efficiency. Functional feed additives not only improve poultry health and growth performance but also significantly influence meat quality traits, including oxidative stability, tenderness, water-holding capacity and nutritional composition. The major effects of different additives on meat quality characteristics are summarized in Table 3.

## 6. Reduction of Antibiotic Use and Meat Safety

### 6.1. Lower Residue Risks and Consumer Safety

One of the greatest public health benefits of transitioning to antibiotic-free production systems through the use of functional feed additives is the reduced risk of antimicrobial resistance and antibiotic residue accumulation in poultry products [20], which results in the elimination or dramatic reduction of antibiotic residues. Antibiotic residues have been found in both the liver and breast tissues of processed poultry, and some reports have indicated levels that were higher than the maximum recommended residue limits. [20]. However, the levels will differ according to the antibiotic compound, the type of tissue, the method of analysis, the period of withdrawal and the regulatory standards applied. When broilers are fed functional feed additives instead of antibiotics, there are no residues or very low levels in edible tissues [101]. Avoiding the use of antibiotics may also minimize the risk of allergic response, genetic mutations, damage to bone marrow and disruption of normal human gut microbiota in consumers [18]. Monitoring investigations of antibiotic-free poultry supply chains have shown that, when used appropriately, functional feed additives can support the production of residue-free poultry products that comply with regulatory and food safety requirements.

### 6.2. Microbial Safety and Pathogenic Bacterial Control

Pathogenic bacteria and antimicrobial-resistant organisms are a major route of transmission in the poultry food chain and can lead to human infection and the spread of resistance genes [68]. The antimicrobial properties of probiotics are attributed to their ability to produce bacteriocins and exert competitive exclusion against pathogenic microorganisms [38]. Organic acids and phytogenics decrease the number of pathogenic bacteria and maintain beneficial microorganisms [113]. In the case of *Salmonella* infection, birds fed on a diet containing combinations of probiotics and organic acids showed improved survival and decreased bacterial translocation [82]. Essential oils of oregano and other plants have been shown to have a 98% inhibition efficacy against *E. coli* strains [68]. Although these antimicrobial effects are not as potent as antibiotics, they are not associated with the development of resistance as they act on multiple target sites and disruption mechanisms [5].

### 6.3. Antimicrobial Resistance Gene Reduction and Dissemination Prevention

**Figure 2:**
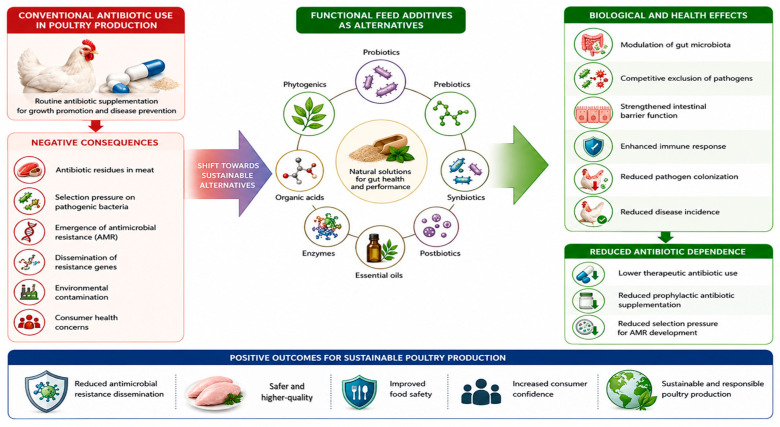
Role of functional feed additives in reducing antibiotic dependence and mitigating antimicrobial resistance dissemination in poultry production systems.

Animal agriculture is considered a major contributor to the global challenge of antimicrobial resistance (AMR) due to the extensive use of antibiotics in livestock production systems [114]. Poultry production systems that use functional feed additives can reduce selective pressure associated with antibiotic use, thereby limiting the persistence and spread of antimicrobial resistance genes [5]. Antibiotic resistance gene (ARG) abundance in manure and the surrounding environment gradually decreases as farms move toward antibiotic-free systems [115]. The frequency of genetic resistance determinants associated with tetracyclines, sulfonamides and β-lactams, the antibiotics most commonly used in poultry production systems, is expected to decline as the selective pressure from antibiotic use is reduced [34]. Functional feed additives help reduce antibiotic dependence by improving gut health, enhancing disease resistance and suppressing pathogenic microorganisms, thereby lowering the selective pressure that drives antimicrobial resistance development. The relationship between functional additives, reduced antibiotic use and the mitigation of antimicrobial resistance is summarized in Figure 2.

### 6.4. Regulatory Compliance and Market Certification

Antibiotic use in animal production is increasingly being regulated across many countries through stricter policies, withdrawal periods, and compliance requirements aimed at ensuring food safety and minimizing antimicrobial resistance [116]. Functional feed additives (FFAs) allow for easy compliance with the increasingly demanding regulatory requirements and for market access to premium products free of antibiotics [5]. The “Raised without Antibiotics,” “No Antibiotics Ever” and organic certification programs also mandate documentation of practices that are compatible with functional additive use [5]. Good documentation and proven methods for functional additives facilitate regulatory inspection and consumer trust in product claims [101]. In addition, compliance with antibiotic restrictions helps producers avoid regulatory penalties and market access limitations that are increasingly enforced in developed markets.

## 7. Consumer Perception and Market Acceptance

### 7.1. Consumer Awareness of Antibiotic-Free Meat

In developed countries, the awareness of consumers about the use of antibiotics in poultry farming and its health implications has significantly grown [16]. Consistent surveys have shown that a large proportion of consumers are interested in consuming antibiotic-free meat due to its health benefits and would like to buy it when available; e.g., a consumer perception study reported that 50% of respondents considered antibiotic-free and hormone-free meat products healthier than non-labeled products, while 63.74% had purchased such products within the previous 12 months [117]. Awareness regarding antibiotic resistance associated with the consumption of animal-based foods is not uniform and tends to be lower in several developing regions, particularly among populations with lower educational attainment and socioeconomic status [118]. Educational campaigns and labelling have been shown to be effective in raising consumer awareness of the risks of antibiotic resistance and its link to food-producing animals [119]. Consumer willingness to purchase antibiotic-free poultry reflects the fact that the market fundamentals for producer transition to alternative production systems are strong.

### 7.2. Perception of Natural Feed Additives

The attitudes of consumers towards “natural” feed additives, such as probiotics, prebiotics and plant-based compounds, are still overwhelmingly positive in various markets [120]. The use of functional additives that are either “natural” or specifically “plant-based” aligns with present-day consumer demand for minimally processed foods and “clean labels” free of synthetic chemicals [16]. However, a significant research gap still exists regarding the scientific mechanisms and long-term effectiveness of these additives, even while many consumers continue to perceive “natural” products as automatically superior [120]. Information on the scientific facts behind the use of functional additives would help consumers make informed choices. Further, third-party certification and transparency regarding the additives, including their sourcing and composition, would further enhance consumer confidence in products that contain additives.

### 7.3. Willingness to Pay Premium Prices

Consumers’ willingness to pay premiums for antibiotic-free poultry with documented functional additive supplementation is a key market driver that will support the economic viability of production transitions [16]. Numerous consumer surveys have found that large proportions of consumers are willing to pay 10–30% more for certified antibiotic-free meat [5]. This price premium is linked to the consumer valuation of health benefits, the perceived superior quality, and personal values on sustainable and responsible food production. Antibiotic-free and organic poultry is selling at premium shelf positioning and retail prices in many developed markets and has proven market acceptance [27]. The premium pricing advantages associated with functional-additive-based production systems can substantially offset the additional costs of additive supplementation for producers.

### 7.4. Labeling, Transparency and Trust Factors

Feed additives and safety certifications are critical factors in consumer purchasing decisions and trust [121]. Consumers are more confident in products that are clearly labeled as “antibiotic-free,” products that contain functional additives, and those that are certified by third parties [5]. On the other hand, products that are not transparent or do not have supporting claims are met with skepticism and less likely to be purchased [121]. Research on the effectiveness of labeling shows that consumers who are given more information about production methods and additives are more likely to feel good about the product and justify higher prices for it [121]. But discrepancies in labelling and greenwashing (claims that are not substantiated as being sustainable) erode consumer confidence. Standardized labelling systems, third-party verification, and transparency would increase market integrity and consumer trust in antibiotic-free products.

### 7.5. Regional Differences and Geographic Variation

**Figure 3:**
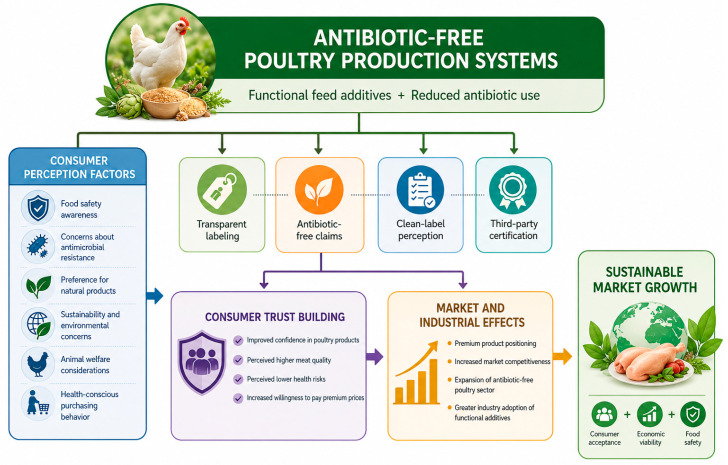
Factors influencing consumer perception, purchasing behavior, and market acceptance of antibiotic-free poultry meat produced using functional feed additives.

Consumer perceptions of the importance of antibiotic-free poultry and natural feed additives show significant geographic differences, which are associated with cultural values, regulatory environments and perceptions of disease burden [27]. European consumers, influenced by the early ban on antibiotics and high food safety awareness, have a special preference for antibiotic-free products [5]. Antibiotic-free poultry products are increasingly gaining acceptance in North American markets; however, a substantial segment of price-sensitive consumers still prioritizes affordability over health-related attributes [17]. Some producer and consumer segments in developing countries, where the burden of infectious disease remains high and biosecurity infrastructure is still weak, consider antibiotics necessary risk-management tools and therefore moderate demand for antibiotic-free products [27]. Regional differences require regionally appropriate marketing, educational and policy strategies for transitioning to functional additive-based systems. Consumer acceptance of antibiotic-free poultry products is influenced by several interconnected factors, including food safety awareness, transparency in labeling, sustainability concerns, perceptions of animal welfare and trust in natural production systems. The major determinants influencing consumer perception and market acceptance are illustrated in Figure 3.

## 8. Economic and Industrial Considerations

### 8.1. Cost-Effectiveness and Economic Feasibility

The economics of changing from antibiotic- to functional-additive-based poultry production systems is one of the key factors in influencing adoption rates. The cost of functional feed additives can vary significantly, ranging from probiotics and prebiotics to essential oils and specialized formulations, which can be much more expensive [122]. When the economic factors are analyzed in detail, however, well-designed functional additive programs create positive ROI due to improved feed conversion ratio, lower mortality, better meat quality, higher premiums and lower medication expenses [122]. A controlled trial comparing different categories of feed additives revealed that nano-zinc supplementation produced the highest economic profit (INR 165,100; approximately USD 1954) along with the greatest benefit–cost ratio (2.62), demonstrating its clear economic viability in broiler production systems [122]. Slightly smaller absolute production gains were observed with enzyme- and probiotic-based systems, but they still delivered good economic returns. The economic benefits of functional additives will be even more apparent when premium market prices for antibiotic-free products are introduced [5].

### 8.2. Scalability and Commercial Farm Implementation

For commercial-scale implementation of functional feed additive systems, standardized formulations, reliable supply chains, effective quality assurance and farmer training are key to ensuring efficacy across different production environments [5]. Large farms with integrated production and feed milling can benefit from optimal cost control and consistency by formulating and incorporating additives in-house [26]. Medium- and small-scale producers rely on commercial premix suppliers and need technical assistance to formulate and administer appropriate diets [26]. Large-scale implementation of integrated Focus Farm Management systems incorporating functional feed additives has been successfully demonstrated in 20,000-bird commercial operations, resulting in measurable improvements in production performance and economic returns [123]. Challenges associated with large-scale implementation include variability in additive quality among suppliers, potential interactions between nutrients and feed additives that require careful formulation, and the need for farmer education regarding the proper dosage and application of these additives [5]. The proven commercial success on varying farm sizes and in different geographic locations, however, suggests that scalability challenges are not insurmountable.

### 8.3. Challenges for Producers and Infrastructure Requirements

Although functional feed additives are considered as alternatives to antibiotics, there are many challenges for producers who are moving away from conventional systems. Disease pressure can be effectively managed in high-biosecurity commercial production systems; however, traditional management systems with inadequate biosecurity and confinement practices may face greater disease-related production challenges [27]. Farmers who have been used to “antibiotic insurance” by giving routine prophylactic treatments need to use advanced health management strategies based on biosecurity, vaccination, environmental control and careful monitoring [119]. In developing countries, small- and medium-scale producers may not have the technical knowledge, record keeping systems or veterinary support to implement evidence-based alternatives [27]. Also, the cost of functional additives may be a problem for producers with narrow profit margins in competitive markets [25]. Proper feed storage, handling, and quality control are essential to prevent additive degradation and maintain their efficacy. Policy support, technical assistance programs and farmer education programs that are specific to the needs of different farmer are necessary to address these challenges [26].

## 9. Sustainability Implications

### 9.1. Reduced Environmental Impact and Waste Management

Poultry production systems with functional feed additives have a significant impact on environmental sustainability in several ways. Decreases in the anti-microbial residues excreted in poultry manure reduces the environmental load of anti-microbial residues and impact on non-target microorganisms in soil and water ecosystems [26]. Research on antibiotic residue levels in poultry manure shows significantly higher levels in conventionally managed (antibiotic-treated) poultry manure than in those that are not treated with antibiotics [124]. Probiotics and organic acids modulate intestinal fermentation, which will lead to decreased ammonia and methane emissions from the poultry manure and reduce atmospheric pollution and greenhouse gas production [25]. Improved feed conversion ratio (FCR) leads to better nutrient utilization efficiency, which decreases the total amount of feed produced, the application of fertilizers in the fields and the emissions of greenhouse gasses from agricultural systems used for feed production [53]. The reduced production cycle is associated with better health and growth performance, which reduces the amounts of environmental resources used over the entire production process for each unit of meat produced.

### 9.2. Production Efficiency and Resource Optimization

The overall production efficiency is enhanced by functional feed additives, which act in several ways to improve resource efficacy. Better feed conversion ratios help to decrease overall feed usage and production costs [50]. The improved nutrient bioavailability and absorption efficiency enhance the nutritional value of dietary ingredients, enabling the substitution of high-cost refined ingredients with more sustainable, locally sourced alternatives [70]. A lower incidence of disease and reduced dependence on antibiotics lead to lower production losses and costs [119]. The use of functional additives in breeder flocks improves reproductive performance, reducing the number of replacement birds needed [26]. All these efficiencies together lower per-unit production costs and resource use, improving the economic sustainability of poultry production and lowering absolute resource use.

### 9.3. Contribution to Sustainable Livestock Systems

Sustainable livestock production systems are fundamentally based on practices that are compatible with long-term environmental health, animal welfare and food security [26]. Poultry production with the use of a functional feed additive is a significant contribution to sustainability goals. The “tragedy of the commons” associated with antibiotic use is minimized in these systems, as the individual benefits of antibiotic-driven growth promotion are outweighed by the broader societal risks linked to the development of antimicrobial resistance [26]. Functional-additive-based systems help maintain the efficacy of antibiotics in animal therapeutic use and contribute to long-term food security and public health by reducing reliance on antibiotics. The integration of functional additives into a holistic farm management system can facilitate circular nutrient flow, as it can enhance the quality of manure and minimize chemical residues [50]. These systems align with consumer expectations for environmentally responsible food production and offer market opportunities for premium positioning, thereby improving the economic viability of smaller-scale and regenerative farming systems [70].

## 10. Challenges and Limitations

### 10.1. Variability in Additive Efficacy and Inconsistent Results

Much research has been done to show the benefits of using functional feed additives, but there is large variation in efficacy results when using them in the field in different production situations [119]. This variability is due to several interacting factors, such as additive formulation, strain selection, dosage, feed matrix composition and environmental conditions [26]. For instance, the efficacy of probiotics is highly strain-dependent and relies on the ability of the strains to survive in the proventriculus and gizzard, adhere to the intestinal wall and metabolize in the avian digestive system, which cannot be guaranteed in all commercial products [53]. The prebiotic effectiveness depends on the composition of intestinal microbial communities, host genetics, carbohydrate composition of the diet and production stress factors [50]. The phytogenic efficacy varies depending on the season because of the different plant materials used, extraction techniques and the stability of bioactive compounds during storage and feed preparation [70]. This variation makes it difficult to apply it consistently in the field and to maintain farmer confidence in the value of the additive.

### 10.2. Dosage Optimization and Standardization Gaps

There are no standard dosage recommendations for most functional feed additives, and considerable differences exist between research protocols, commercial products and practical applications [119]. Concentrations used in research studies are usually chosen for maximum effectiveness under experimental conditions and may not be the most cost-effective or practical field concentrations [26]. Optimal doses are likely to be dependent on production type, bird age, health status, diet composition and environmental stressors, but commercial products rarely give context-specific advice [53]. This lack of standardized recommendations introduces regulatory uncertainty, farmer confusion over correct application and the risk of both under-dosing (inefficacy) and over-dosing (wasted expense or potential adverse effects) [50]. There is still a lack of industry standardization, which is still fragmented by additive class and geographic region.

### 10.3. Interaction Effects with Diet and Management Factors

The efficacy of functional feed additives is determined by interactions with the general diet composition, farm management, environmental conditions and genetic backgrounds of production birds [70]. Such interactions are not fully understood and cannot be used to predict results in different production situations [119]. For instance, the effectiveness of organic acids is highly dependent on the protein-to-mineral ratio in the diet, processing conditions of the feed and water quality parameters [26]. The efficiency of enzymes is influenced by the substrate, the feed composition and the thermal stability during feed manufacturing and storage [53]. The effect of probiotics is highly dependent on the history of antibiotic use, dietary fiber content, stocking density and environmental temperature [50]. The results of highly controlled research environments do not always apply in a less controlled commercial farm environment with a wider range of management practices due to these multi-factorial interactions.

### 10.4. Lack of Regulatory Standardization and Quality Control

Unlike pharmaceutical interventions and growth-promoting antibiotics, there is no standardization of regulatory frameworks for functional feed additives [70]. Each regulatory jurisdiction worldwide has different criteria for approval, quality criteria, labeling requirements and efficacy documentation thresholds [119]. The regulatory framework of many jurisdictions permits additives to be marketed based on traditional use or limited efficacy data, which results in inconsistencies in the product quality and benefits claimed [26]. The level of active ingredient, microbial viability (in probiotics), bioactive compound identity along with their concentration (in phytogenics) and product stability are not consistent among manufacturers [53]. There is still limited third-party testing and certification, which means that there is still an information imbalance between producers and users [50]. Such gaps in standardization pose problems for farmers’ decision making, regulatory compliance throughout markets and the development of uniform evidence bases for efficacy and safety statements.

## 11. Future Perspectives and Innovation Opportunities

### 11.1. Precision Nutrition and Personalized Feed Formulation

Precision nutrition approaches, which tailor feed formulations to specific flock characteristics, production phases and health statuses, will be a key focus of future poultry production systems [125]. This shift takes advantage of the progress in genomics, microbiomics and metabolomics to define individual nutritional needs optimized for specific genetic lines and production systems [119]. Precision systems rely on functional feed additives, which can be used to intervene at specific points to correct specific nutritional deficiencies or imbalances in the microbiota [26]. Dynamic feed formulation adjustments based on real-time monitoring of flock health, growth and feed efficiency parameters can maximize the additive benefits while minimizing unnecessary supplementation [126]. Precision nutrition combined with functional additives has the potential to significantly increase resource efficiency, decrease dependence on antibiotics and improve product quality compared to traditional fixed-formula nutrition [127].

### 11.2. Artificial Intelligence and Advanced Feed Formulation

Artificial intelligence (AI) and machine learning technologies are increasingly being explored for optimizing feed formulation and precision poultry nutrition. These methods can help to process large volumes of data concerning flock performance, feed composition, environmental conditions, gut health and the effects of additives on flock performance to aid in evidence-based nutritional decisions [50,70]. Initial research indicates that AI-powered models could be beneficial for enhancing feed efficiency, forecasting disease risks and fine-tuning additive mixes within specific production settings [109]. But the use of AI-based systems in commercial poultry production is still limited and needs to be further validated in different production settings. Predictive models are highly dependent on the quality, quantity and standardization of production data. Furthermore, implementation costs, technical skills, data integration and scalability issues can limit adoption, especially in small- and medium-scale production systems [26]. Thus, more extensive research and field testing are needed to ensure that AI feed formulation can be applied on a practical scale in commercial poultry farming.

### 11.3. Combination Strategies and Synergistic Applications

Today, combinations of complementary functional additives are more and more recognized as having better effects than single-ingredient methods [53]. The combination of probiotics with prebiotics (synbiotics) is more effective due to the synergistic effect of the two components working together in complementary ways [50]. The use of probiotics or phytogenics with organic acids is effective in enhancing the anti-microbial properties and in establishing beneficial microbiota [76]. The use of essential oils in combination with enzyme supplementation improves digestion and antimicrobial defense [119]. The strategic co-combination of various functional classes of additives that are optimized for the respective production problems will lead to improved performance compared to the use of single-ingredient additives [26]. The future will see further innovation to identify the best combinations of additives, to understand the synergistic effect of these combinations and to create standardized combination products that are best suited to specific production phases and problems.

### 11.4. Functional Meat Branding and Market Differentiation

**Figure 4:**
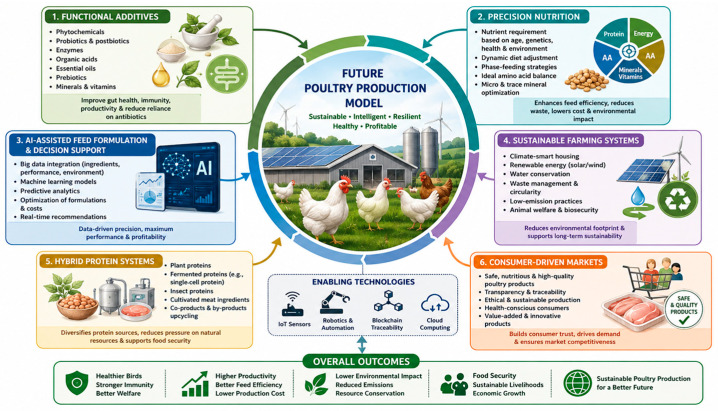
Integrated future poultry production model for sustainable, AI-driven and precision-based poultry systems.

Market opportunities for premium meat products produced using functional-additive-based systems are expanding as the adoption of functional feed additives increases and accumulating evidence continues to demonstrate their positive effects on meat quality [53]. Functional poultry meat branding strategies can highlight the nutritional benefits, food safety attributes, environmental sustainability, animal welfare attributes and antibiotic-free production credentials [53]. As consumers are willing to pay higher prices for naturally produced, antibiotic-free meat, there are economic incentives for producers to adopt functional additive systems [76]. Clear labeling and certification schemes that confirm functional additives, production methods and proven benefits will help to build consumer confidence and differentiate the market [119]. The expanding market opportunities are likely to stimulate investment in functional additive research, product development and producer education, thereby promoting greater innovation and adoption within the poultry industry. Despite the promising potential of functional feed additives, several scientific, economic and regulatory challenges remain before their universal adoption can be achieved. Current limitations and future research opportunities are summarized in Table 4. Meanwhile, future poultry production systems are expected to integrate precision nutrition, artificial-intelligence-assisted feed formulation, sustainable farming practices and functional feed additives to optimize productivity, meat quality, environmental sustainability, and consumer acceptance. A conceptual overview of these integrated future production systems is presented in Figure 4.

## 12. Conclusions

The trend of decreasing antibiotic use in poultry production has become a critical global priority because of growing concerns about antimicrobial resistance, antibiotic residues in edible tissues and the sustainability of intensive livestock systems. While antibiotics have historically helped to improve growth performance, feed efficiency and disease prevention, their use as a routine and subtherapeutic basis in animal production raised serious public health and regulatory concerns. Consequently, the poultry industry is now moving towards production systems based on safe, sustainable and consumer-acceptable alternatives. Of these, functional feed additives have received significant scientific and commercial interest as potential alternatives and/or supplements to traditional antibiotic approaches.

Available data indicate that functional feed additives, such as probiotics, prebiotics, synbiotics, phytogenics, organic acids, enzymes, essential oils, minerals, vitamins and postbiotics, have a positive effect on poultry performance by several biological mechanisms. These include the modulation of the intestinal microbiota, strengthening of the gut barrier, activation of immune responses, activation of digestive enzyme activity, inhibition of pathogenic microorganisms and reductions in oxidative stress. These processes, in turn, allow birds to more effectively absorb nutrients, support gut health and deal with environmental or disease challenges without overreliance on antibiotic use.

These additives not only improve flock health and productivity, but also enhance the quality of poultry meat, a crucial aspect in contemporary markets. Better feed efficiency and metabolic health are often correlated with better carcass yield, optimized postmortem pH decline, better water holding capacity, less drip and cooking loss, better tenderness and better stable color characteristics. Furthermore, the use of antioxidant additives (phytogenics and essential oils) helps to reduce lipid oxidation, which also increases shelf life and helps maintain flavor during storage. There are also some indications of improved nutritional composition, such as increased protein retention and stability of fatty acids. These quality improvements are very relevant to processors, retailers and consumers looking for consistent and premium poultry products.

On a food safety basis, reduced antibiotic use significantly reduces the risk of residues in meat and decreases the selection pressure of antimicrobial-resistant bacteria in the poultry production system. The transition therefore not only helps to ensure safer food products but also supports wider One Health objectives that connect animal, human and environmental health and protection. Moreover, lower excretion of antibiotics in manure could limit the spread of resistance genes and pharmaceuticals into the environment.

The success of these alternative production systems is determined by consumer perception. Today’s consumers are more likely to think of antibiotic-free poultry meat as better for their health, better quality, better for the animals and better for the environment. With growing awareness, consumers are willing to pay higher prices for “antibiotic-free,” “naturally raised” or “sustainably produced” poultry products. Thus, the strategic deployment of functional feed additives can provide opportunities for producers to differentiate in the market, build their brand and secure access to premium retail segments. Trust and proof of added value will be crucial and will rely on transparent labeling, third-party certification and science-based communication.

Although these encouraging results have been achieved, there are still a number of hurdles to overcome before universal adoption can be realized. The effectiveness of functional feed additives depends on the birds’ genetic makeup, hygiene on the farm, stocking density, feed composition, environmental stress, dosage and quality of the additives. Field responses are inconsistent, there are no uniform regulations, and costs are higher in the initial stages, which may prevent adoption, especially by small- and medium-scale producers. Functional additives should not be considered as a direct replacement for antibiotics, but as part of a comprehensive health management program that includes effective biosecurity, vaccination, welfare-based housing, nutrition management and regular flock monitoring.

Precision poultry nutrition, microbiome-based interventions, omics technologies, and artificial-intelligence-assisted feed formulation will help drive future progress in this area. These can be used to determine the best combination of additives, optimize feed cost and customize feeding programs for different production situations. Combinations of probiotics, enzymes, organic acids and phytogenic compounds might provide greater and more consistent effects than individual additives. Ongoing studies are also required to confirm long-term performance in commercial use and to develop internationally accepted standards for efficacy, safety and labelling.

One of the most effective and scientifically supported strategies to reduce dependence on antibiotics in poultry production, while maintaining productivity, quality of meat, consumer confidence and environmental sustainability, is the use of functional feed additives. They can be integrated successfully into modern poultry systems and contribute to the development of a safer, more resilient and economically viable poultry industry that can responsibly meet future global protein needs.

## Figures and Tables

**Table 1:** Classification of functional feed additives.

Additive Type	Examples	Primary Mechanism	Major Benefits
Probiotics	*Lactobacillus*, *Bacillus*	Competitive exclusion	Gut health [39], FCR [40]
Prebiotics	Mannan-oligosaccharides (MOS), fructo-oligosaccharides (FOS)	Selective fermentation	SCFA production [41]
Synbiotics	Probiotic + prebiotic	Synergistic microbiota modulation	Immunity [42]
Phytogenics	Oregano, thyme	Antioxidant/antimicrobial	Meat quality [43]
Organic acids	Butyrate, acetate	Lower gut pH	Pathogen reduction [44]
Enzymes	Xylanase, protease	Improve digestion	Nutrient absorption [45]
Essential oils	Rosemary oil	Improve body weight gain	Improve production [46]
Postbiotics	Fermentation metabolites	Immune modulation	Safe alternative [47]

**Table 2:** Effects of functional feed additives on poultry growth performance and health parameters.

Additive	Species/Strain	Inclusion Level	Major Findings	Reference
Probiotics	Cobb 500 broilers	10^9^ CFU/g	Improved FCR	[81]
Synbiotics	Broilers	0.75 g/kg	Increased BWG	[82]
Organic acids	Ross 308	1%	Lower mortality	[83]
Phytogenics	Cobb 500 broilers	150 mg/kg diet	Improved antioxidant status and gut health	[84]
Essential oils	Ross 308 broilers	100 mg/kg	Enhanced feed efficiency and microbial balance	[85]
Enzymes	Broilers	Commercial enzyme supplementation	Improved nutrient digestibility	[86]
Postbiotics	Cobb 500 broilers	0.3%	Improved immune response and gut integrity	[87]
Vitamins/Minerals	Broilers	Zinc (32–40 mg/kg) Vitamin E (40 to 80 IU/kg feed)	Enhanced growth performance, antioxidant defense and immunity	[88,89,90]

**Table 3:** Effects of functional feed additives on poultry meat quality characteristics and shelf life stability.

Additive	Meat Quality Parameter	Observed Effect	Proposed Mechanism	References
Essential oils	Lipid oxidation	Reduced TBARS values and improved shelf life stability	Antioxidant and free radical scavenging activity	[106]
Probiotics	Tenderness	Improved tenderness and sensory acceptability	Enhanced nutrient utilization and muscle metabolism	[107]
Prebiotics	Water-holding capacity (WHC)	Increased WHC and reduced drip loss	Improved gut health and nutrient absorption	[108]
Phytogenics	Color stability	Improved oxidative stability and color retention	Presence of phenolic antioxidant compounds	[109]
Synbiotics	Sensory quality	Improved flavor and overall acceptability	Modulation of gut microbiota and metabolism	[110]
Organic acids	Meat safety	Reduced pathogenic bacterial load	Lower gastrointestinal pH and antimicrobial effects	[44]
Postbiotics	Texture and juiciness	Reduced cooking loss and shear force	Improved muscle integrity and water retention	[111]
Vitamins/minerals	Oxidative stability	Enhanced antioxidant capacity of meat	Increased antioxidant enzyme activity	[112]

**Table 4:** Current limitations and future direction in poultry production.

Current Limitation	Impact	Possible Solution	Future Direction
Variable efficacy	Inconsistent outcomes	Standardization	Precision nutrition
High cost	Reduced adoption	AI feed optimization	Smart formulation
Regulatory gaps	Market inconsistency	Global standards	Harmonized policies

## Data Availability

No new data were created or analyzed in this study. Data sharing is not applicable to this article.

## Abbreviations

The following abbreviations are used in this manuscript:

ADG	Average Daily Gain
AGPs	Antibiotic Growth Promoters
AI	Artificial Intelligence
AMR	Antimicrobial Resistance
ARGs	Antibiotic Resistance Genes
BWG	Body Weight Gain
CFU	Colony-Forming Units
FCR	Feed Conversion Ratio
FOS	Fructo-Oligosaccharides
IEL	Intraepithelial Lymphocytes
IgA	Immunoglobulin A
IgG	Immunoglobulin G
IgM	Immunoglobulin M
IU	International Units
MCFAs	Medium-Chain Fatty Acids
MDA	Malondialdehyde
MEAs	Microecological Agents
MOS	Mannan-Oligosaccharides
MRLs	Maximum Residue Limits
NAE	No Antibiotics Ever
NSP	Non-Starch Polysaccharides
PFAs	Phytogenic Feed Additives
ROI	Return on Investment
ROS	Reactive Oxygen Species
RWA	Raised Without Antibiotics
SCFA	Short-Chain Fatty Acids
sIgA	Secretory Immunoglobulin A
TBARS	Thiobarbituric Acid Reactive Substances
WHC	Water-Holding Capacity
